# A Comparative Study of N-Acetyl Cysteine, Rosuvastatin, and Vitamin E in the Management of Patients with Non-Alcoholic Steatohepatitis: A Randomized Controlled Trial

**DOI:** 10.3390/ph18050650

**Published:** 2025-04-29

**Authors:** Amr Y. Zakaria, Rehab Badawi, Hasnaa Osama, Mona A. Abdelrahman, Asmaa M. El-Kalaawy

**Affiliations:** 1 Pharmacy Practice (Clinical Pharmacy) Department, Faculty of Pharmacy, Horus University-Egypt, New Damietta 34517, Egypt; ahamza@horus.edu.eg; 2 Tropical Medicine and Infectious Diseases Department, Faculty of Medicine, Tanta University, Tanta 31527, Egypt; rehab.elsheshtawy@med.tanta.edu.eg; 3 Clinical Pharmacy Department, Faculty of Pharmacy, Beni-Suef University, Beni Suef 62514, Egypt; dr_mona_2008@yahoo.com; 4 Pharmacology Department, Faculty of Medicine, Beni-Suef University, Beni Suef 62511, Egypt; asmaa.hussein@med.bsu.edu.eg

**Keywords:** NASH, steatosis, fibrosis, N-acetyl cysteine, rosuvastatin, vitamin E

## Abstract

**Background**: Non-alcoholic steatohepatitis (NASH) is characterized by increased production of proinflammatory cytokines, fibrosis, and hepatocyte apoptosis. This study aimed to assess the efficacy of N-acetyl cysteine (NAC), rosuvastatin (RSV), and vitamin E (VE) in patients with NASH. **Methods**: A double-blinded, parallel, randomized, controlled study was conducted and registered on clinicaltrials.gov (Identifier: NCT06105060), involving 135 NASH participants, who were divided into three groups: the control group (group 1), consisting of patients receiving standard therapy VE at a dosage of 400 IU twice daily. In the treated group (group 2), patients were administered NAC at a dosage of 1200 mg twice daily, while treatment (group 3) received RSV at a dosage of 20 mg once daily. FibroScan^®^ examination of liver tissue and fibrosis scores, along with tests for liver aminotransferases, lipid profile, glycemic parameters, and renal and hepatic functions, were assessed before and after six months of treatment. **Results**: The analyzed groups demonstrated a significant reduction in steatosis and lipid peroxidation (*p* < 0.05). The NAC group demonstrated greater anti-inflammatory and anti-apoptotic effects compared to the RSV group, although this difference was not significant in the control group. NAC is conceded as the only significant antifibrotic agent in liver stiffness measurement (LSM), biological marker findings, and non-invasive liver fibrosis scores (*p* < 0.05), in addition to its improvement of several metabolic parameters and health-related quality of life. **Conclusions**: Patients receiving NAC demonstrated safety and efficacy in enhancing steatosis, fibrosis, and metabolic parameters, representing a novel strategy in the management of NASH.

## 1. Introduction

Non-alcoholic fatty liver disease (NAFLD) is one of the widely growing signs of metabolic syndrome on a global scale. Non-alcoholic steatohepatitis (NASH) is a severe form of NAFLD characterized by significant triglyceride accumulation, hepatic injury, excessive production of proinflammatory cytokines, and apoptosis of hepatocytes [1,2]. In severe cases, NASH can promote the development of hepatic cirrhosis, hepatic cell carcinoma, the need for liver transplantation, and ultimately mortality [3]. Recently, NAFLD is more common in men than in women (31% vs. 16%), and the prevalence rises with age, from less than 20% in those under 20 to over 40% in those over 60, according to Egypt’s country-specific data. The prevalence rate is 15.8% in children and adolescents (6–18 years old) [4,5]. In addition, NASH places a substantial financial strain on countries [6].

The precise etiology of the development from NAFLD to NASH remains uncertain and is likely attributable to a multifaceted hypothesis resulting from the interplay of environmental factors, patient metabolism, insulin resistance (IR), gut flora imbalance, genetic factors, free radicals, mitochondrial dysfunction, altered production of adipokines and cytokines, and endoplasmic reticulum. Nevertheless, several health problems, including obesity, hypertension, and diabetes, have been identified as factors that boost the likelihood of its manifestation [7].

Herein, identifying pre-symptomatic people who are at risk of developing NAFLD/NASH would be the most effective approach to facilitate earlier intervention for the condition. NASH is frequently mischaracterized as a condition devoid of symptoms [8]. Despite the increasing prevalence of NASH, there remains a dearth of efficacious non-invasive diagnostic tools for this condition [9].

The pathophysiology of NAFLD is known to entail oxidative stress (OS), characterized by an imbalance between prooxidants and antioxidants. This imbalance leads to hepatic damage and is responsible for the development of NASH. Consequently, we have the potential to analyze the pathways and measure the metabolites associated with this process [10]. Clinical studies have found that the serum level of malondialdehyde (MDA) is elevated in NAFLD, indicating a significant increase in lipid peroxidation [11]. Oxidative stress is indicated by elevated levels of MDA in the blood, serving as a reliable marker for lipid peroxidation [12]. Furthermore, fibrosis may result from MDA’s capacity to stimulate hepatic stellate cells (HSC) to produce collagen [13].

The transition from NAFLD to NASH is characterized by the activation of inflammatory processes, which may be assessed by measuring inflammatory markers and mediators such as tumor necrosis factor (TNF-α) and the NOD-like receptor-associated protein 3 (NLRP3) inflammasome [14]. Since the NLRP3 inflammasome is indispensable for the processing of the principal proinflammatory cytokines and can lead to the progression of NAFLD into NASH [15], new evidence from experimental models shows that NLRP3 inflammasome-associated medications and inhibitors can effectively improve NASH [15,16]. Moreover, TNF-α remains a valuable tool for monitoring the inflammatory metabolic status and the advancement of liver damage [17]. The progression of a chronic inflammatory response results in the accumulation of extracellular matrix (ECM) components in the liver, consequently forming fibrous scar tissue [18]. Fibroblast growth factor-21 (FGF21) is a hormone that modulates critical metabolic pathways; peroxisome proliferator-activated receptors-α (PPAR-α) is essential for regulating FGF21 expression [19]. Obesity, elevated body mass index (BMI), and steatosis are positively linked with FGF21 levels. Evidence suggests elevated FGF21 levels may serve as a defense mechanism against lipotoxicity [20].

**Figure 1:**
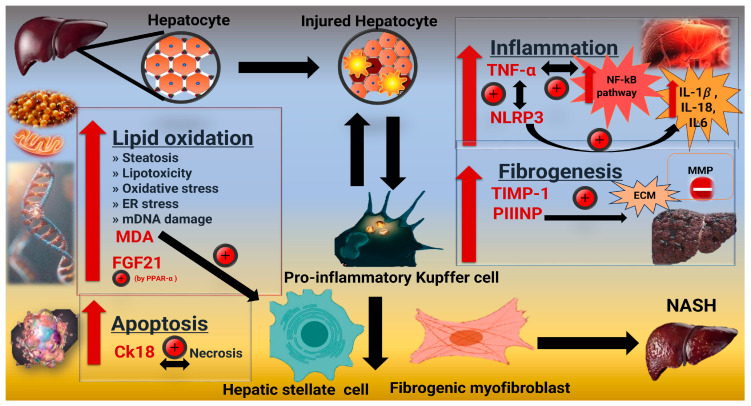
A summary of the mediators involved in the pathogenesis of NASH progression. NAFLD elevates serum malondialdehyde (MDA) levels, signifying a substantial rise in lipid peroxidation and oxidative stress (OS) as evidenced by lipid oxidation. Increased MDA, activates hepatic stellate cells (HSC) to synthesize collagen. Peroxisome proliferator-activated receptors-α (PPAR-α) regulate the adipokine hormone known as fibroblast growth factor-21 (FGF21) which is increased by steatosis. The activation of the Nuclear Factor Kappa B (NF-κB) pathway by reactive oxygen species (ROS) facilitates the assembly of the NOD-like receptor-associated protein 3 (NLRP3) inflammasome and enhances the expression of tumor necrosis factor-α (TNF-α), which activates macrophages and Kupffer cells, thereby promoting inflammation. The NLRP3 inflammasome activates mature caspase-1, resulting in the production of mature cytokines interleukins (IL-1β, IL-18, IL-6) that initiate inflammation and pyroptosis, contributing to the development of NAFLD to NASH. The formation and accumulation of various extracellular matrix (ECM) components impede the degradation of fibrous tissue due to increased synthesis of Tissue inhibitor of metalloproteinases 1 (TIMP-1) and decreased production of fibrolytic matrix metalloproteinases (MMPs), both by hepatic stellate cells/myofibroblasts and Kupffer cells/macrophages. N-terminal pro-peptide of procollagen type III (PIIINP) serves as a circulating biomarker for extracellular matrix remodeling in liver fibrogenesis. The full-length form of cytokeratin-18 (CK18) fragments is released from cells experiencing necrosis. CK18 (human cytokeratin- 18), ECM (extracellular matrix), ER (endoplasmic reticulum), FGF21 (fibroblast growth factor-21), MDA (malondialdehyde), MMPs (matrix metalloproteinases), NASH (Non-alcoholic steatohepatitis), NF-κB (Nuclear Factor Kappa B), NLRP-3 (NOD-like receptor protein 3 inflammasome), PPAR-α (Peroxisome proliferator-activated receptors-α) PIIINP (pro-collagen type III N-peptide), TIMP-1 (Tissue inhibitor of metalloproteinase-1), TNF-α (Human tumor necrosis factor α), + (stimulator), and − (inhibitor).

Fibrosis is a pathophysiological process that occurs in advanced liver disease. It is characterized by the abnormal growth of fibrous connective tissue in the liver, indicating the progression of NAFLD [21]. During fibrogenesis and fibrinolysis, fragments of ECM are released into the bloodstream. By monitoring the serum level of these molecules, one can assess the extent of liver fibrosis [22]. Tissue inhibitor of metalloproteinases 1 (TIMP-1) regulates ECM alteration in the liver via the activity of matrix metalloproteinases (MMPs) [23]. TIMP-1 is utilized to identify advanced fibrosis associated with NASH [24]. The elevation of the N-terminal pro-peptide of procollagen type III (PIIINP) may be ascribed to increased collagen production and degradation. Therefore, the levels of PIIINP in the bloodstream have been identified as a crucial biomarker for detecting liver fibrosis without the need for invasive procedures [25]. Patients with NASH were shown to have elevated levels of cytokeratin-18 (CK18) fragments [26]. This suggests that CK18 fragments can distinguish NASH from simple steatosis. The CK18 full-length form is released from cells undergoing necrosis [27]. Determining CK18 levels independently predicts the presence of NASH [28]. The mediators involved in the pathogenesis of NASH are summarized in (Figure 1).

To date, numerous clinical trials are presently evaluating novel molecules for the management of NASH [29]. Prior studies have shown that reducing energy consumption and engaging in regular exercise over a period of six to twelve months can enhance metabolic parameters and decrease inflammation and steatosis [30].

Vitamin E (VE) is an essential fat-soluble antioxidant that has been proven to enhance the functioning of immune cells [31]. PIVENS trial demonstrated that a 2-year intervention with 800 IU of alpha-tocopherol (*α*-TOH) in non-diabetic patients with NAFLD led to a significant decrease in steatohepatitis relative to placebo [32]. Additionally, significant decreases in hepatic transaminase levels were noted. However, there was no significant change in the level of fibrosis, thus VE is endorsed by the American Association for the Study of Liver Diseases (AASLD) [33].

Rosuvastatin (RSV) is a hydrophilic, highly selective 3-Hydroxy-3-Methylglutaryl-Coenzyme (HMG-CoA) reductase inhibitor, exhibiting a significant level of selectivity for hepatocytes in comparison to various non-hepatic cells, including cultured human skeletal muscle cells [34]. Furthermore, RSV experiences minimal metabolism via the hepatic Cytochromes P (CYP) system exhibits moderate systemic bioavailability, and possesses a relatively extended elimination half-life. Based on these criteria, RSV signifies an advancement in the efficient use of the pharmacologic properties within the statin class [35]. The use of RSV has been linked to elevated levels of glutathione synthase (GSS), glutathione peroxidase (GPx), glutathione reductase (GR), and glutamylcysteine synthetase [36]. According to a previous study, RSV displays dose-dependent antioxidant and anti-inflammatory effects [36]. RSV may positively influence the resolution of NASH by activating the phosphatidylinositol 3-kinase (PI3K)/protein kinase B (AKT)/mammalian target of rapamycin (mTOR), Nuclear factor erythroid 2–related factor (2 Nrf2), Heme oxygenase (HO-1), called (PI3K/Akt/Nrf2/HO-1) pathway [37,38]. In addition, RSV diminishes OS through various antioxidant mechanisms, including the reduction in NADPH oxidase, suppression of endothelial nitric oxide synthase (eNOS) uncoupling, upregulation of antioxidant enzymatic defense mechanisms, and blocking hydrogen peroxide-induced DNA damage [36].

N-acetyl cysteine (NAC) is generally considered safe and well-tolerated, even at large dosages. The liver then directs most of the liberated cysteine toward the biosynthesis of glutathione (GSH) [39]. Nevertheless, NAC has the potential to decrease increased levels of alanine aminotransferase (ALT) in patients with NASH [40]. Furthermore, administering NAC may enhance glucose tolerance and increase peripheral insulin sensitivity [41].

We aimed to assess and compare the effects of RSV, VE, and NAC in Egyptian patients with NASH. Through a six-month study of the degree of fibrosis improvement without exacerbation of NASH, or NASH resolution without deterioration of fibrosis and steatosis, and study the enhancement of biochemical markers associated with diagnostic scores and the extent of steatosis, lipid peroxidation, inflammation, liver fibrosis, and apoptosis.

## 2. Results

### 2.1. Socio-Demographic, Anthropometric Measurements Assessments Among the Studied Groups Before and After Treatment

**Figure 2:**
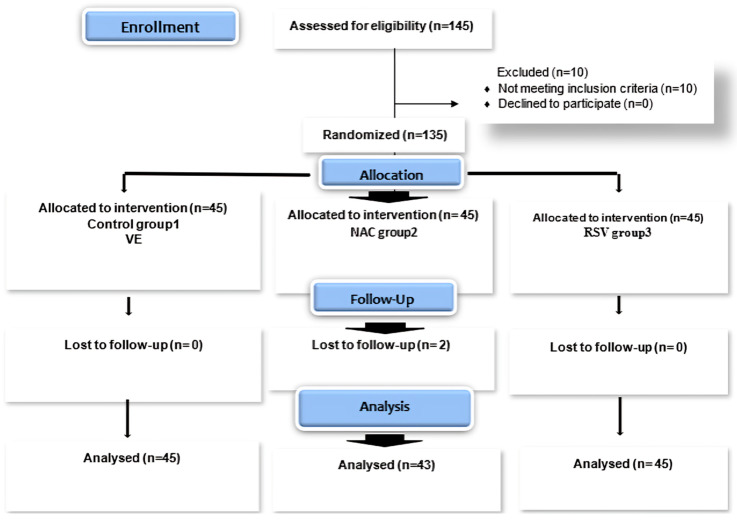
Flow diagram of patient progress through phases of a randomized trial.

The study included 135 participants diagnosed with NASH through physical examinations, elevated liver enzymes, ultrasonography, and FibroScan^®^ assessments. In addition to the Fibroscan-aspartate aminotransferase (FAST) score, the Fibrosis-4 Index (FIB-4), the Fibrotic NASH Index (FNI), and combination of aspartate aminotransferase, homeostasis model assessment, and cytokeratin 18 (MACK-3) were evaluated. Ten patients were excluded from the study due to failure to comply with the inclusion criteria. A total of 45 participants were involved and evenly distributed between the control and treatment groups (grps). Two patients were lost to follow-up in the second group (Figure 2).

Patients undergoing NAC and RSV treatments for six months had a decrease in lean body weight and BMI (*p* < 0.05). Concurrently, the VE group exhibited no significant differences. Waist circumference (WC) and hip circumference (HC) within grp 2 demonstrated a significant reduction (*p* = 0.001). Waist–hip ratio (WHR) exhibited a noteworthy decrease in groups 2 and 3, with reductions of approximately 2.37% (*p* = 0.001) and 0.75% (*p* = 0.032), respectively. There was an improvement in the waist–stature ratio (WSR) within grps 2 and 3, with a significant decrease of 5.06% (*p* = 0.001) and 1.58% (*p* = 0.019), respectively. Upper mid-arm circumference (MUAC) fell significantly in grp 2 by 4.22% (*p* = 0.001) and in grp 3 by 2.03% (*p* = 0.002). The differences among and within the studied grps and demographic data are shown in (Table 1).

### 2.2. Steatosis and Fibrosis Degree and Studied Markers Among the Studied Groups Before and After Treatment

#### 2.2.1. Steatosis and Fibrosis

The current study demonstrated steatosis grades among patients with NASH. In control, grp 1, a statistically significant decrease, after 6 months, in the mean value of steatosis, approximately 6.05% (*p* = 0.017), 14.2% (*p* = 0.001) in treated grp 2, and 7.52% (*p* = 0.004) in grp 3. A significant difference was observed exclusively post-treatment between the NAC and RSV grps (*p* = 0.029). This study examined fibrosis grades in individuals with NASH. Group 2 demonstrated a sole statistically significant reduction in the mean value of fibrosis by approximately 12.5% (*p* = 0.001). Besides substantial differences between grp 2 and 3 post-intervention (*p* = 0.028), no statistical significance was observed after the administration of VE and RSV treatments and subsequent evaluations.

#### 2.2.2. Scoring System

The FAST and FNI scores analysis revealed a significant reduction in the mean across the three grps before and after the intervention (*p* = 0.001). The significance of the FIB-4 score demonstrated a decrease in the percentage of mean values within treatment grps 2 and 3, showing reductions of 27.03% (*p* = 0.001) and 8.03% (*p* = 0.04), respectively. MACK-3 demonstrated a significant reduction in mean values in treatment grp 2 by 23.8% (*p* = 0.001), whereas a significant increase was recorded in grp 3 by 6.46% (*p* = 0.046), besides the absence of a significant difference within grp 1 in the two later scales. The differences within and among the study grps before and after the intervention are illustrated in (Table 2).

### 2.3. Laboratory Values of the Studied Groups Before and After Treatment

Groups 1, 2, and 3 exhibited a significant reduction in ALT levels of 6.25% (*p* = 0.01), 16.67% (*p* = 0.002), and 6.67% (*p* = 0.02), respectively. Group 2 displayed a significant reduction in aspartate aminotransferase (AST) levels of 16.13% (*p* = 0.005). AST/ALT showed no significant differences, except for a 23.26% increase in the ratio in grp 3 (*p* = 0.047). Group 2 demonstrated a significant reduction in hemoglobin A1c (HbA1c) by 1.90% (*p* = 0.004), along with a decrease in fasting blood glucose (FBG) by 3.6% (*p* = 0.009), body insulin by 8.6% (*p* = 0.009), and the homeostatic model assessment for insulin resistance (HOMA-IR) by 11.11% (*p* = 0.001). Regarding the lipid profile, treatment grp 2 exhibited significant reductions in serum cholesterol, triglycerides (TGs), low-density lipoprotein (LDL), very low-density lipoprotein (VLDL), cholesterol/high-density lipoprotein (HDL), and LDL/HDL by 5.41%, 9.33%, 6.49%, 20.59%, 12.42%, and 15.58%, respectively, while HDL increased by 7.9% (*p* = 0.001). In grp 3, there was a significant decrease by 20.07%, 24.18%, 24.31%, 26.19%, 30.29%, and 34.98%, respectively, while HDL increased by 14.95% (*p* = 0.001). The intra- and inter-group differences in laboratory values of the studied groups are illustrated in (Supplementary Table S1).

### 2.4. Biomarkers of Inflammation, Fibrosis, and Apoptosis of the Studied Groups Before and After Treatment

**Figure 3:**
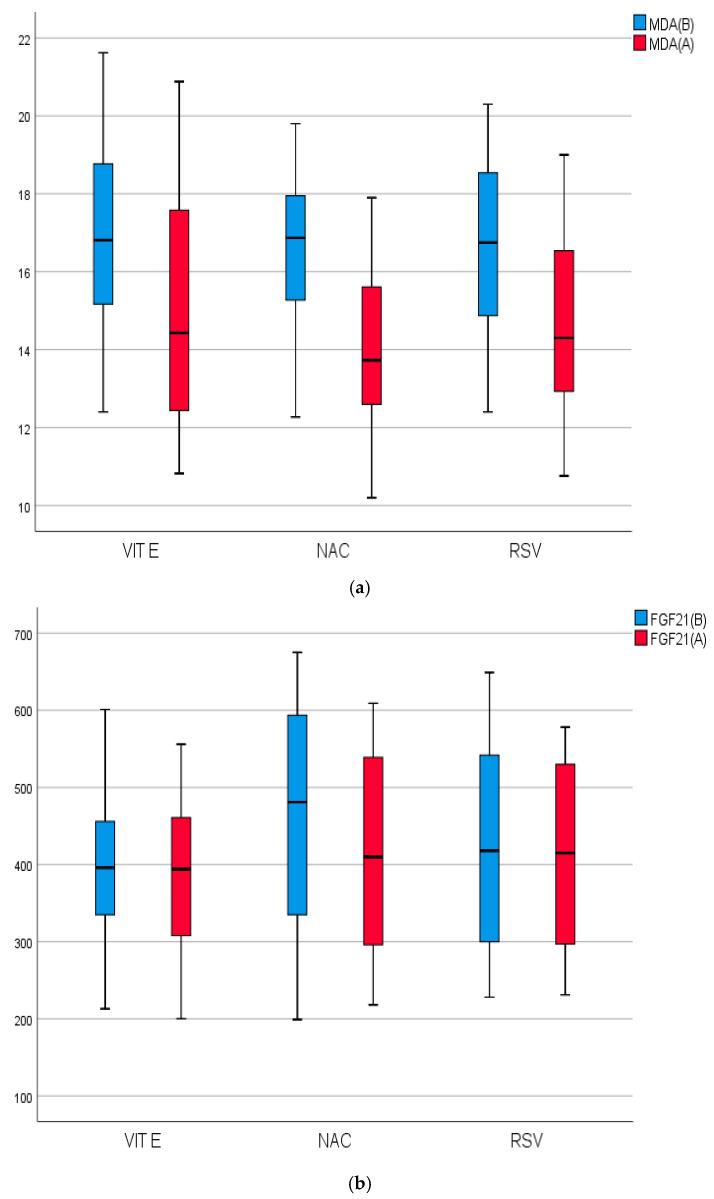

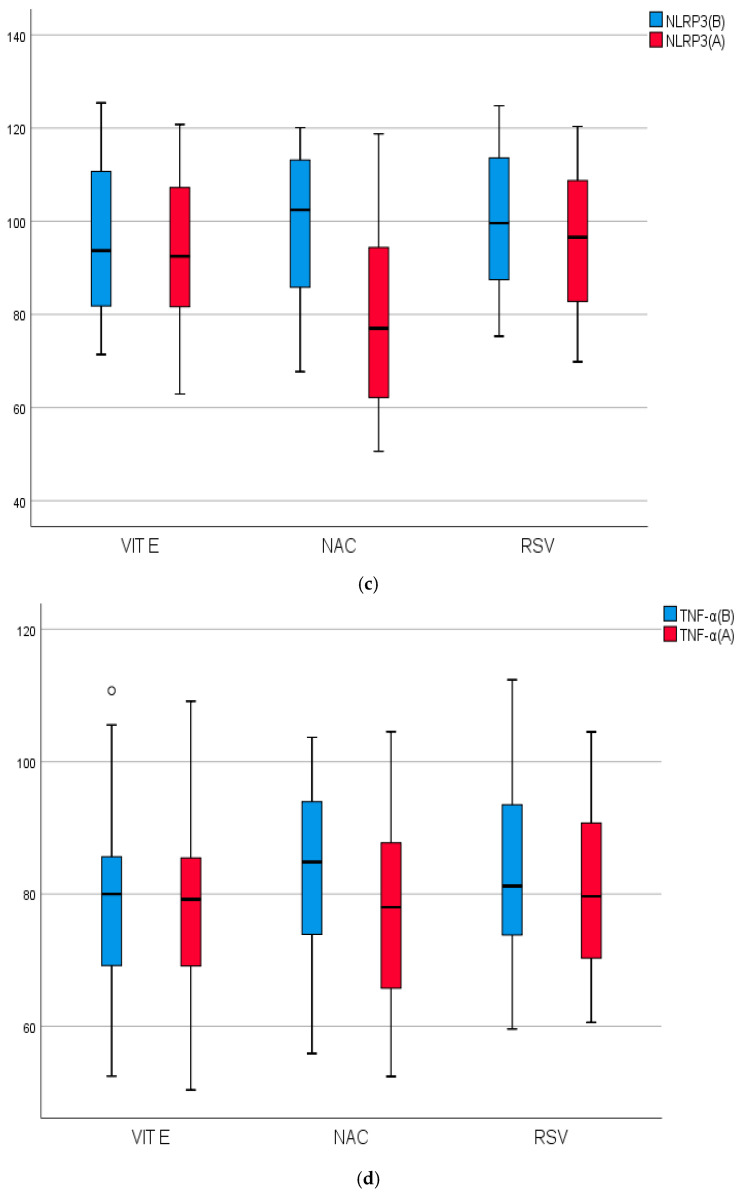

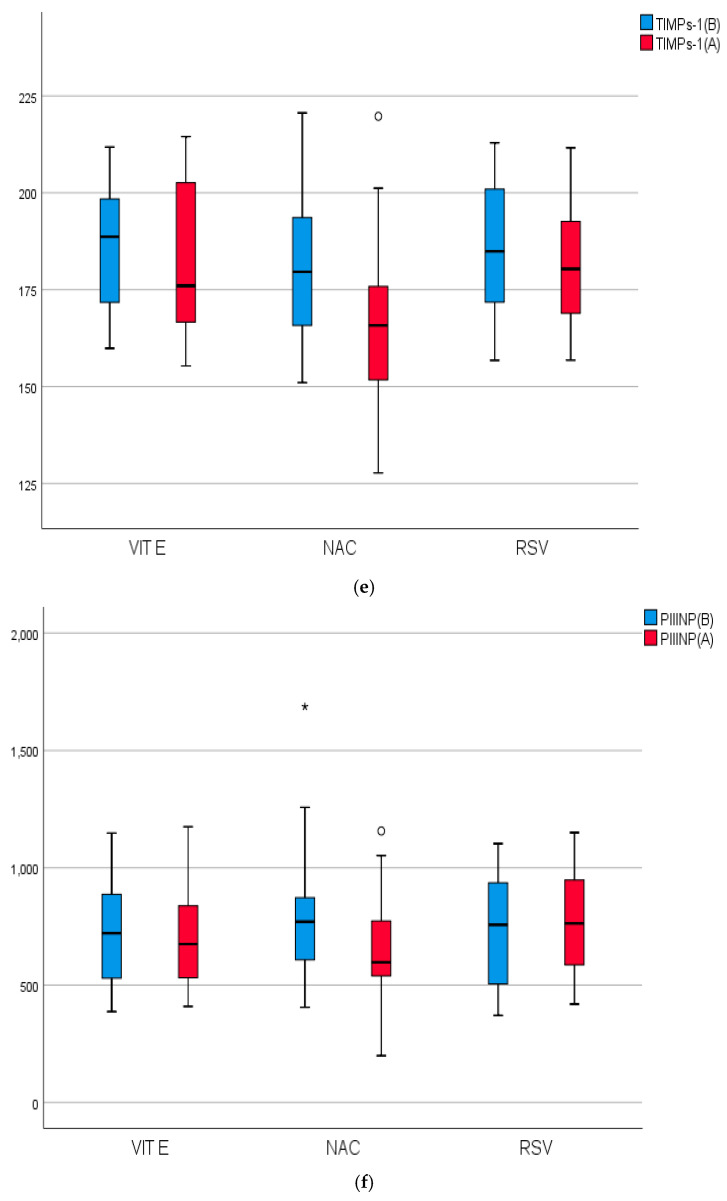

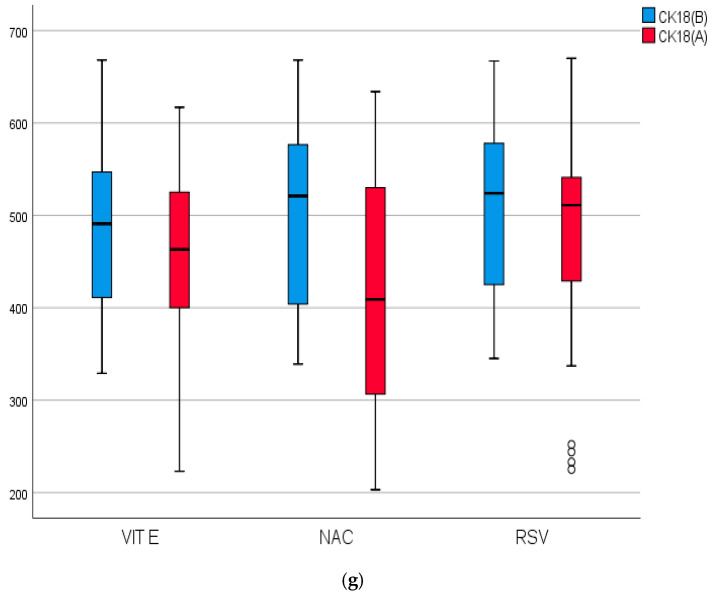
(**a**) Biomarkers of lipid peroxidation of the studied groups before and after treatment, Control group 1: Vitamin E (VE); group 2: N-acetyl cysteine (NAC); group 3: Rosuvastatin (RSV); Before intervention (B); after intervention (A); MDA: malondialdehyde. (**b**) Biomarkers of adipokine hormone FGF21 involved in lipid peroxidation of the studied groups before and after treatment. Control group 1: vitamin E (VE); group 2: N-acetyl cysteine (NAC); group 3: rosuvastatin (RSV); before intervention (B); after intervention (A); FGF21: Fibroblast growth factor-21. (**c**) Biomarkers of inflammation of the studied groups before and after treatment. Control group 1: vitamin E (VE); group 2: N-acetyl cysteine (NAC); group 3: Rosuvastatin (RSV); before intervention (B); after intervention (A); NLRP3: NOD-like receptor-associated protein3 inflammasome. (**d**) Biomarkers of inflammation of the studied groups before and after treatment, control group 1: vitamin E (VE). Group 2: N-acetyl cysteine (NAC); group 3: rosuvastatin (RSV); before intervention (B); after intervention (A); TNF-α: tumor necrosis factor; (o): extreme values. (**e**) Biomarkers of fibrosis of the studied groups before and after treatment. Control group 1: vitamin E (VE); group 2: N-acetyl cysteine (NAC); group 3: rosuvastatin (RSV); before intervention (B); after intervention (A); TIMP-1: tissue inhibitor of metalloproteinases- 1; (o): extreme values. (**f**) Biomarkers of fibrosis of the studied groups before and after treatment. Control group 1: vitamin E (VE); group 2: N-acetyl cysteine (NAC); group 3: rosuvastatin (RSV); before intervention (B); after intervention (A); PIIINP: N-terminal pro-peptide of procollagen type. (*/o): extreme values. (**g**) biomarkers of apoptosis and necrosis of the studied groups before and aftertreatment. Control group 1: vitamin E (VE); group 2: N-acetyl cysteine (NAC); group 3: rosuvastatin (RSV); before intervention (B); after intervention (A); CK18: cytokeratin-18; (o): extreme values.

Regarding MDA, grps 1, 2, and 3 exhibited significant decreases in mean values by 11.90% (*p* = 0.006), 15.97% (*p* = 0.001), and 11.51% (*p* = 0.001), respectively. Serum NLRP-3 mean levels in grps 2 and 3 showed significant reductions of 20.04% (*p* = 0.0001) and 4.09% (*p* = 0.007), respectively. No noteworthy variations emerged within grp 1. TNF-α mean levels in grps 2 and 3 exhibited significant reductions of 7.49% (*p* = 0.001) and 2.71% (*p* = 0.002), respectively. No significant differences were observed within grp 1. The mean levels of FGF21, TIMPs-1, and PIIINP displayed a substantial decline exclusively in grp 2, with decreases of 10.78% (*p* = 0.024), 8.68% (*p* = 0.0001), and 15.37% (*p* = 0.001), respectively. The serum level of CK18 exhibited a significant decrease of approximately 16.54% (*p* = 0.001) in grp 2, as well as a decrease of about 5.13% (*p* = 0.04) in grp 3. Intra- and inter-group variations among the studied grps before and after interventions are shown in (Figure 3a–g).

### 2.5. Assessment of Quality of Life Short-Form 36 (SF-36) Domains and Adverse Events Version 5.00 (CTCAE) Among Studied Groups Before and After Treatment

**Figure 4:**
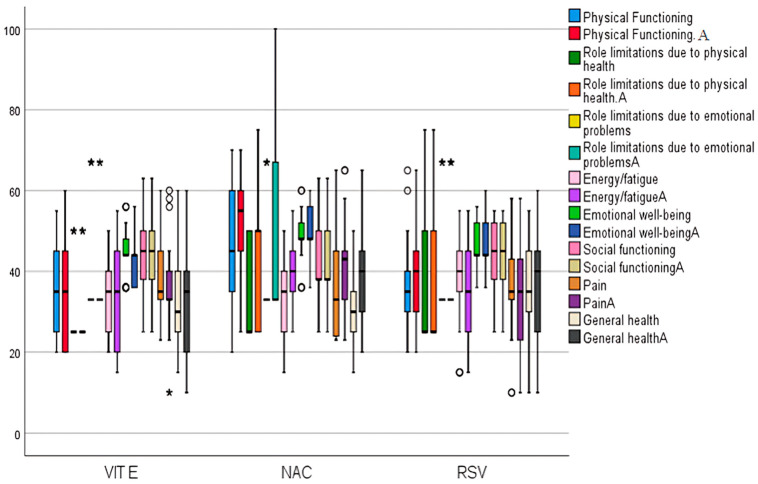
Quality of life Short-Form 36 (SF-36) domains among studied groups before and after treatment. Control group 1: vitamin E (VIT E); group 2: N-acetyl cysteine (NAC); group 3: rosuvastatin (RSV). After intervention (A); (*, o): extreme values.

There was a significant improvement in physical functioning domains by 22.22% (*p* = 0.001); in addition, the improvements in role limitations due to physical health, and role limitations due to emotional scale were exclusively within treatment grp 2 (*p* < 0.05). Furthermore, significant improvements in the energy/fatigue scale were observed in grp 1 (*p* = 0.001) and grp 2 (*p* = 0.001). On the emotional well-being scale, there were significant differences within grps 1 and 3 (*p* < 0.05). Statistically significant improvements in the pain scale were noted in group 2 (*p* = 0.001), whereas noteworthy worsening occurred in intragroup 1 (*p* = 0.001) and 3 (*p* = 0.006). The general health scale showed statistically significant improvements in grp 1 (*p* = 0.049) and grp 2 (*p* = 0.001), as shown in (Figure 4). Concerning clinical symptoms and adverse events, version 5.00 (CTCAE) indicates that no serious adverse events (GRADE 3, 4, or 5) were observed in the examined groups, as shown in (Supplementary Table S2).

## 3. Discussion

The majority of patients with NASH remain asymptomatic; however, symptomatic individuals often exhibit elevated levels of the liver function enzymes ALT and AST [42]. At the beginning of treatment, our results indicated that patients screened by FibroScan^®^ [43] for steatosis and fibrosis exhibited significantly increased levels of CK18 [44] and mild to moderate elevations in hepatic liver enzymes [45]. This supports the prospective role of inflammatory mediators in the pathophysiology and progression of NASH, consistent with the studies by Maher M et al., Kalas M et al., and Newsome, Philip N et al. [43,44,45].

After six months of treatment, our results indicated that control grp 1 exhibited a significant reduction in steatosis and ALT levels, corroborating the work of Chee, Nicholas et al., which demonstrates that VE improves liver function [46]. In the study of Jawad Fairooz et al., VE alone exhibited a significant decrease in steatosis scores following 12 weeks of therapy [47]. Our clinical findings suggest the beneficial effect of NAC in reducing liver injury and disease progression, highlighting its potential therapeutic role in clinical applications. These findings are consistent with the results of Khoshbaten et al., indicating that a three-month supplementation of NAC (1200 mg/day) can decrease ALT levels and spleen size in patients with NAFLD, leading to an improvement in fatty infiltration due to NAC’s capacity to inhibit lipid peroxidation propagation, which is evident in its role in preventing the onset of NAFLD [48]. A recent consistent study by Tsai, Ching-Chou et al. demonstrated that NAC therapy mitigates hepatic steatosis and apoptosis produced by a high-fat diet (HFD) [49]. In RSV grp 3, the results regarding steatosis are in line with those of Kargiotis, Konstantinos et al. [50]. Another way that RSV reduces steatosis is by influencing the homeostasis of peroxisome proliferator-activated receptors (PPARs), which in turn reduces hepatic steatosis in NAFLD mice [51].

Indeed, the results of liver stiffness measurement (LSM) fibrosis and prognosis fibrosis scales indicate that the potential impact of control VE group 1 on fibrosis is intricate. Certain study suggests that VE may provide preventive benefits against OS and hepatic injury; nonetheless, its impact on fibrosis remains contentious. A trial performed by Yakaryilmaz et al. demonstrated that VE enhanced liver enzyme levels in individuals with NASH, corroborating our findings; nevertheless, it did not significantly influence fibrosis or necroinflammation [52]. A randomized trial suggested that VE may enhance liver histology in patients with NASH; however, the association between VE and fibrosis remains inconclusive [53], which is consistent with our findings. Conversely, in NAC, grp 2 is more effective in reducing hepatic fibrosis. The results are consistent with the findings of the study by Mazo, Daniel FC, indicating that NAC appears to exert its effects by reducing OS and influencing fibrogenic pathways, including the modulation of MMP and transforming growth factor β-1 (TGF-β-1) [54]. RSV grp3 exhibits significant fibrotic activity, with a MACK-3 score increase of 6.46% (*p* = 0.046). According to Vargas, J. I. et al., there have been incidences of liver fibrosis linked to statin use, suggesting that although these medications can benefit some people, they can also harm others [55]. The disparity is striking, proving that NAC is superior in reducing steatosis and fibrosis in patients with NASH.

Regarding biological markers for lipid peroxidation, the grp 1 exhibited a significant reduction in serum MDA levels after six months, with a decrease of 11.90% (*p* = 0.006). Elevated amounts of non-esterified fatty acids resulted in elevated levels of MDA in peripheral blood mononuclear cells. However, prior administration of VE mitigated the accumulation of MDA levels [31]. Admittedly, In the meta-analysis conducted by Zheng, Sai-Hua et al., supplementing with antioxidant vitamins like VE was linked to a reduction in plasma MDA levels in women diagnosed with endometriosis [56]. In our study, the NAC group showed the most decrease in serum MDA after six months compared to the other study arms, with a decline of 15.97% (*p* = 0.001). Since NAC may scavenge free oxygen radicals, remove reactive oxygen species (ROS)-induced cell damage, and boost glutathione S-transferase activity that restores damaged targets in critical cellular components [57]. Interestingly, the study conducted by C. C. Xu et al. found that the levels of free radicals were reduced and the activities of antioxidant enzymes were raised in the gastrointestinal tract of piglets treated with NAC. These changes suggest that NAC treatment may help restore the imbalance in gut redox and reduce OS [58]. In addition, patients whose NAC was administered orally experienced a 2.5-fold decrease in MDA [59,60]. Our study revealed a notable decrease in serum MDA levels in the RSV group after six months, with a reduction of 11.51% (*p* = 0.001). RSV is a statin with anti-inflammatory and antioxidant properties; it does this by activating the Nrf2 and HO-1 signaling pathways [61]. The Nrf2 protein and its downstream proteins have a crucial role in avoiding chemical damage and OS in liver cells [62]. Nrf2 regulates the expression of more than 100 genes, such as HO-1, and various proteins that protect cells and enzymes that counteract oxidative stress, such as superoxide dismutase (SOD) and glutathione S-transferase (GST). This regulation occurs when Nrf2 binds to certain regions of DNA known as antioxidant response elements, resulting in cellular protection. The Nrf2/HO-1 signaling pathway has the ability to significantly decrease the formation of ROS in mitochondria and control the overall functionality of mitochondria [63]. This pathway also influences the expression and activity of antioxidant enzymes. Activation of the Nrf2 pathway has demonstrated efficacy in reducing liver damage caused by drugs or xenobiotics [64]. Some believe that the PI3K/Akt pathway plays a significant role in regulating Nrf2 and other antioxidant response components that rely on OS defense. The expression and phosphorylation of the PI3K protein were both markedly enhanced by RSV treatment. Research by Yeh et al. demonstrated that RSV protected atrial myocytes from tachycardia-induced damage by activating the PI3K/Akt/Nrf2/HO-1 pathway [38].

In the context of the inflammatory process, serum NLRP3 in the NAC group demonstrated a significant reduction of 20.04% (*p* = 0.0001) throughout the treatment period in our study. ROS can cause thioredoxin-interacting protein (TXNIP) to separate from thioredoxin (TRX) and attach to NLRP3, leading to the activation of NLRP3. NAC removes ROS and inhibits the NLRP3 and pyroptosis in NASH by targeting the residual oxygen consumption (ROX)-TXNIP axis [15]. The study conducted by Zhen Luo et al. confirms that NAC effectively reduced the levels of inflammatory cytokines in both mothers and placentas by inhibiting the NLRP3 inflammasome. Additionally, it decreased the expression of autophagy proteins and boosted the activity of the extracellular signal-regulated kinase (ERK) and Akt/mTOR signaling pathways. Surprisingly, the intake of NAC resulted in modifications to the fecal microbial populations and metabolites, including fecal levels of Prevotella and Clostridium cluster XIVa [65]. The study by Xiaopeng Liu et al. demonstrated that NAC can reduce NLRP3 expression in macrophages, resulting in decreased interleukin (IL-18) production [66]. In grp 3, serum NLRP-3 levels decreased significantly by 4.09% (*p* = 0.007). RVS reduced the expression levels of NLRP3, caspase-1, interleukin-1β, and Gasdermin D N-terminal domains. Luo et al. demonstrated that RSV’s suppression of NLRP3 aligns with its protective effects against diabetic cardiomyopathy [67].

Secondly, TNF-α levels in grp 2 exhibited a reduction of 7.49% (*p* = 0.001). NAC may alter the TNF-α receptor and diminish the binding capacity of TGF-β1 to the type III transforming growth factor receptor (TβRIII) beta-glycan [39]. Furthermore, NAC inhibits the movement and activation of the transcription nuclear factor-kappa B (NF-κB) within the nucleus, which is important for controlling the expression of genes associated with inflammation [68]. The study conducted by Abdelhafez, D. et al. suggests that the use of bone marrow–mesenchymal stem cells in combination with antioxidants (NAC and ascorbic acid) may offer a potential treatment for acute pancreatitis. This treatment has the potential to inhibit TNF-α, IL 1β, and NF-κβ [69]. In grp 3, the TNF-α value decreased significantly by 2.71% (*p* = 0.002). RSV has been demonstrated to impact the adaptive immune response, as indicated by a reduction in the expression of TNF-α and interferon gamma (IFN-γ) in lymphocytes in individuals diagnosed with acute coronary syndrome [70]. Chen, Weijian et al. performed a study on the effects of TNF-α stimulation. Furthermore, RSV effectively prevents the activation of cell pyroptosis and senescence caused by TNF-α. [71].

Thirdly, FGF21 demonstrated a significant reduction of 10.78% (*p* = 0.024) exclusively in NAC. The study by Murali, Chaya N et al. highlighted the necessity of exploring the relationship between NAC and FGF21. Potential indicators of cysteine’s influence may encompass growth differentiation factor 15 (GDF15), FGF21, blood cysteine levels, and the resolution of lactatemia and liver failure [72]. The improvement of fibrosis and steatosis in patients with NASH coincides with a reduction in FGF21 levels. Our study represents a groundbreaking investigation into the direct relationship between the intervening medicine and FGF21.

In the context of fibrosis biomarkers, TIMPs-1 exhibited a significant reduction of 8.68% (*p* = 0.0001) solely in the NAC group. Previous studies indicate that NAC may directly inhibit the activation of NF-kB or the promoter of MMP-9. This subsequently inhibits the activity of MMP-9. It is probable that NAC directly inhibits the gelatinolytic activity of MMP-2 [73,74]. The study conducted by Aslantaş, Eda Ezgi et al. revealed that the mRNA expression of TIMP-1 and TIMP-2 was reduced at 24 h when Ca (OH)2 and NAC were administered [75]. The administration of NAC alone effectively inhibited the elevation of MMP-1 and TIMP-1 induced by SiO2 dust, which leads to silicosis fibrosis in rats, by regulating the Nrf2/HO-1 pathway, maintaining the anabolic balance of the extracellular matrix, and obstructing the activation of the ASMase/ceramide signaling pathway [76]. In the NAC group, another biological marker of fibrogenesis, PIIINP, showed a substantial decrease of 15.37% (*p* = 0.001). The study conducted by Yang, Ying-Ying et al. found that there was a reduction in hepatic levels of PIIINP, mRNA of PIIIP, and α-smooth muscle actin (α-SMA). The findings indicate that NAC may possess both direct and indirect anti-fibrogenic properties in rat livers with bile-duct ligation [77]. The findings of Yang, Ying-Ying et al. indicate that the anti-fibrogenic activity of NAC is a significant factor behind the decrease in intrahepatic resistance observed in cirrhosis rats treated with NAC [77].

In the context of the apoptosis process, CK18 exhibited a significant reduction of approximately 16.54% (*p* = 0.001) in the grp 2. According to the study of Gonsebatt, ME et al., NAC greatly reduced the induction of CK18, reducing it by nearly twice [78]. Clinical data on NAC’s protective benefit against drug-induced liver injury is critically discussed by Ntamo, Yonela et al. NAC is believed to diminish keratin-18 and circulating caspase-cleaved CK18 [79]. On the same side, the antioxidant NAC consistently leads to a considerable decrease in the formation of the CK18 protein. This indicates that ROS from the flavoprotein NADPH oxidase enzymes (NOX), particularly NADPH oxidase enzyme 1 (NOX1), which regulates the CK18 protein. This was described in the study carried out by Sattayakhom, A. et al. [80]. In RSV grp3, CK18 showed a notable reduction of around 5.13% (*p* = 0.04) in the RSV group. Consequently, the administration of RSV as the sole treatment for patients with NASH consistently resulted in a decrease in the concentration of IL-6 and CK18. However, these changes did not reach a statistically significant level, according to the study of Aleksandrovich et al. [81]. On the other hand, the study conducted by Kravchenko, LS et al. found that hypolipidemic therapy with RSV in patients with NAFLD for 3 months resulted in a significant drop in CK18 levels by 9% (*p* > 0.05) [82].

In the context of liver and renal function, NAC demonstrated significant improvement in both functions, consistent with the findings of Hatami et al. NAC improved hepatic and renal function. NAC resulted in a notable reduction in Child-Pugh and MELD scores [83,84]. NAC enhances metabolic parameters such as obesity, dyslipidemia, blood glucose, and insulin levels, as evidenced by the studies of Sohouli, Mohammad Hassan, and Liu, Jiajun [85,86]. NAC enhances the quality of life with minimal adverse effects, even at high doses, consistent with the findings of Jayaram, L. et al. and Schwalfenberg, Gerry K. et al. [87].

On the other hand, the RSV group demonstrates significant improvement in dyslipidemia compared to other grps [88] while exhibiting deteriorated glycemic parameters, including HOMA-IR and FBG levels, consistent with the findings of Cheng, Wan-Yin et al. [89]. Liver toxicity is an observed side effect associated with statin treatment [90]. Pre-marketing research studies indicate that RSV may exhibit a reduced potential for hepatotoxicity relative to other statins [91]. The results of Kargiotis, Konstantinos et al. indicate that RSV monotherapy may improve biopsy-proven NASH and resolve metabolic syndrome within 12 months [50], which is consistent with our findings of liver function tests. Consequently, further studies are necessary to investigate any underlying risk of hepatic injury associated with long-term RSV use in this clinical setting.

### The Limitations

The limitations of our study include its single-center design and transient elastography’s restricted capacity to differentiate between early fibrosis stages (F0 and F1). Nevertheless, it has been thoroughly validated for detecting significant fibrosis (≥F2) [92]. Additionally, various factors may restrict the clinical therapeutic use of medication, including treatment adherence, age, gender, nutritional status, disease states, and genetic polymorphisms that can affect the risk of adverse events and treatment resistance. Drug–drug interactions represent a significant variable affecting patient responses to medications [93]. We recommend additional clinical studies with larger sample sizes to corroborate the causality of this observation.

## 4. Materials and Methods

### 4.1. Patients

The recruited patients were from a single center, the outpatient hepatology clinic of the Tropical Medicine and Infectious Diseases Department at Tanta University Hospital, FibroScan^®^, and nutrition units. Patients participating in this study were enrolled from December 2023 to June 2024 based on defined inclusion and exclusion criteria.

The inclusion criteria encompass participants of both genders, age ≥18 years, who have been diagnosed with NASH based on mild to moderate elevation of liver enzymes, ALT, AST, serum aminotransferases (>2, but <5 times upper limit of normal), and ultrasound imaging (confirming the presence of fatty liver in the patients). Moreover, patients were diagnosed with NAFLD through clinical examination, including criteria such as obesity, high BMI, and height. NASH diagnosis using FibroScan^®^ detects the degree of steatosis and fibrosis. Diagnostic accuracy for at-risk NASH (identifying patients who have advanced disease) is by non-invasive scoring such as FAST score, FIB-4, FNI, and MACK-3 scores. Patients with CK18 >240 U/L and stable dietary habits and physical activity patterns.

Conversely, the exclusion criteria encompassed alcohol consumption, viral hepatitis, hemochromatosis, Wilson’s disease, and renal impairment. Furthermore, this investigation specifically eliminated recorded medications that induce steatosis and contraindications of NAC or RSV. In addition, elevated HbA1c levels of 6.5% or above, history or intended bariatric surgery, and patients with additional medical conditions that could increase hepatic enzyme levels, such as pregnancy or heart failure. The study also excluded individuals with a history of breastfeeding, the presence of any condition for FibroScan^®^ contra-indicated, and the patients who declined to participate in or complete the study.

### 4.2. Methods

#### 4.2.1. Study Design

The present study was structured by the Consolidated Standards of Reporting Trials (CONSORT) standard [94]. The study was a 6-month prospective, double-blinded, parallel, randomized, controlled trial. A total of 145 patients were screened, with 135 meeting the eligibility criteria and subsequently enrolled in the study. Participants will be randomly assigned in a 1:1:1 ratio by a neutral researcher utilizing sealed envelopes and assignment codes to three groups. Next, the authors randomly and evenly allocated patients to treatment grps by flipping a coin, with 45 patients in each grp assigned as follows:

- The control grp, grp 1, NASH patients, perceived regular treatment (VE 400 IU^®^; PHARCO-pharmaceuticals), twice daily, for 6 months [95];
- In the treated grp, grp 2, patients received a high dose of NAC, Gemacysteine 300 mg^®^; GEMA-Pharma 1200 mg twice daily, for 6 months;
- In the treated grp, grp 3, patients received RSV; Crestor 20 mg^®^; AstraZeneca: 20 mg/day orally, for 6 months.

Our study aimed to evaluate and compare the preventive effects of VE, NAC, and RSV in Egyptian patients with NASH. The principal goal of this 6-month study will be an enhancement in fibrosis without exacerbation of NASH or resolution of NASH without deterioration of fibrosis and steatosis, with the study being successful if either endpoint was achieved. The secondary aim of this study was the enhancement of biochemical markers associated with steatosis, lipid peroxidation, inflammation, fibrosis, and apoptosis in patients with NASH, in addition to assessing medication adverse effects and quality of life impact.

Before recruitment, approval was obtained from the Ethics Committee, and informed consent was secured from the subjects involved. All patients received the same diet program and were followed up for 6 months at the Nutrition Clinic of Tanta University Hospital. Patients were monitored monthly to assess concession, adverse events, and treatment tolerability.

#### 4.2.2. Ethical Approval

The work has been conducted following the World Medical Association’s code of ethics (Declaration of Helsinki) for human experimentation. The Research Ethics Committee of the University approved the study (APPROVAL CODE 36264PR433/11/23), and it was registered on clinicaltrial.gov (Identifier: NCT06105060). All patients must provide signed written informed consent and agree to comply with the study protocol. The standardized management of NASH is weight control, lifestyle modification, and VE 400 IU twice daily. All patient data will remain private and confidential. Unforeseen risks that arose during the study will be communicated to both patients and the ethical committee without delay.

#### 4.2.3. Anthropometric Measurements

Each patient participating in this study had a thorough evaluation of their medical history, demographic information, and measurements of height and weight. WC, HC, and upper MUAC were assessed, and calculations were performed for WHR and WSR [96,97,98]. Measurements of height and weight were taken using the Smart Lab F500, imported by Modern Pharma International^®^, (Beijing, China). BMI was determined using the following equation: BMI = [Mass (kg)] divided by height (m)^2^] [99].

#### 4.2.4. FibroScan^®^ Examination of the Liver Tissue and Fibrosis Scores

Transient elastography (FibroScan 502 Touch; Echosens, Paris, France) was conducted on a different day to assess steatosis and fibrosis. Participants were instructed to fast for a minimum of 3 h before the diagnostic procedure. Each patient required 10 valid measurements to obtain a controlled attenuation parameter (CAP) score and LSM. The analysis is deemed reliable when it includes at least 10 valid measurements, a success rate of 60% or higher, and IQR/M ≤30% [100].

The FAST score was calculated using [eˆ(−1.65 + 1.07 × In (LSM) + 2.66∗10^−8^ × CAP3 − 63.3 × AST−1)]/[1 + eˆ(−1.65 + 1.07 × In (LSM) + 2.66∗10^−8^ × CAP3 − 63.3 × AST−1)] as carried out by Newsome et al. [101]. The equation determined FIB-4 index: Age (years) × AST [U/L]/(platelets [109/L] × (ALT [U/L])1/2) [102]. FNI = ep/1+ ep: (where *p* = −10.33 + 2.54*ln (AST, U/L) + 3.86*ln (HbA1c, %) − 1.66*ln (HDL, mg/dL) [103]. MACK-3 was computed according to previously reported methods of the study by Boursier et al. [104].

#### 4.2.5. Biochemical Assays

Blood samples were obtained as a baseline before medical intervention and 10–12 h following the administration of the final dosage of the medicine. Blood samples were obtained by extracting 5 mL of serum from each patient. ALT, AST, alkaline phosphatase (ALP), gamma-glutamyl transferase (GGT), and total bilirubin (TBIL), along with serum creatinine, blood urea, uric acid, lipid profiles, complete blood count (CBC), FBG, HbA1c, insulin levels, and creatine phosphokinase (CPK), were quantified following manufacturer protocols [105,106,107,108,109,110,111,112,113,114]. HOMA-IR was calculated by multiplying fasting insulin (in microU/L) by fasting glucose (in nmol/L) and then dividing the result by 22.5 [115]. Biological marker evaluation for lipid peroxidation (MDA), Catalogue No. (BC0020); Solarbio Science^®^, (Beijing, China) was conducted through a colorimetric assay [116]. The kit employs a double-antibody sandwich enzyme-linked immunosorbent assay (ELISA) from Sunredbio, Shanghai Shanghong (SRB) Biotechnology Co., Ltd.^®^ (Shanghai, China) as the manufacturing protocol. TNF-α: Cat No. (201-12-0083) [117], NLRP-3 inflammasome: Cat No. (201-12-5748) [118], FGF21: Cat No. (201-12-1984) [119], human cytokeratin 18-M65 (CK18): Cat No. (201-12-1667) [120], TIMP-1 Cat No. (201-12-1237) [121], and PIIINP: Cat No. (201-12-1354) were quantified [122].

#### 4.2.6. Evaluation of Study Participants’ Adverse Events, and Health-Related Quality of Life

Patients were assessed for health-related quality of life (HRQoL) both before and after intervention with the use of the SF-36 questionnaire [123]. All potential adverse events that may have occurred throughout the intervention period were recorded and evaluated using Version 5.00 (CTCAE) [124]. Participants were deemed nonadherent and excluded from the study if they consumed less than 90% of the study medications or missed a follow-up meeting during any month of the intervention [125].

#### 4.2.7. Statistical Analysis

Data analysis utilized SPSS software, version 26 (SPSS Inc., Chicago, IL, USA, PASW Statistics for Windows version 26). Qualitative data were expressed through numerical values and percentages. Quantitative data were described using median (interquartile range) for non-normally distributed data and mean ± standard deviation (SD) for normally distributed data, following normality assessment via the Kolmogorov–Smirnov test. Chi-Square, Fisher’s exact test., and Monte Carlo tests were used to compare qualitative data between groups as appropriate. Kruskal–Wallis test was used to compare more than 2 studied groups, respectively, for non-normally distributed data. Wilcoxon signed rank test was used to compare more than two studied periods. Paired t test was used to compare 2 paired readings distributed data. One Way ANOVA test was used to compare more than 2 independent groups with the Post Hoc Tukey test to detect pair-wise comparison. The results obtained were evaluated for significance at the *p* < 0.05 level. The sample size for each group was estimated using G Power program version 3.1.9.7, with a power of 90%, an α error of 0.05, and an effect size of 0.728, following the methodology established by Sanyal and Arun J. et al. [126], and validated by the Community, Environmental, and Occupational Medicine Department at the Faculty of Medicine, Tanta University.

## 5. Conclusions

NAC represents a promising therapeutic agent for NASH, attributed to its multifaceted benefits. This approach improves steatosis, fibrosis, and metabolic parameters, indicating a potential new strategy for managing NASH. NAC exhibits superior anti-inflammatory and anti-apoptotic effects relative to RSV or VE, with significant antifibrotic activity evidenced by liver stiffness measurements and non-invasive fibrosis scores. NAC distinctly enhances various metabolic parameters and improves HRQoL with few adverse effects. NAC has shown considerable enhancement in liver and kidney functions. Therefore, NAC is considered an old drug with new applications in the management of NASH.

## Figures and Tables

**Table 1:** Socio-demographic and anthropometric measurements assessments among the studied groups before and after treatment.

		Control Group 1 (VE) *n* = 45	NAC Group 2 *n* = 43	RSV Group 3 *n* = 45	Test of Significance	Intergroup Significance
Age (y)		47.53 ± 10.44	43.26 ± 12.94	49.49 ± 10.1	F = 3.45 *p* = 0.034 *	P1 = 0.081 P2 = 0.409 P3 = 0.01 *
Sex						P1 = 0.296
Female		16(35.6)	20(46.5)	31(68.9)	Mc = 10.38	P2 = 0.001 *
Male		29(64.4)	23(53.5)	14(31.1)	p = 0.006 *	P3 = 0.03 *
Smoking						
Non smoker		28(62.2)	30(69.8)	31(68.9)	Mc = 2.66	P1 = 0.515
Passive		4(8.9)	5(11.6)	2(4.4)	p = 0.616	P2 = 0.651
Current smoker		13(28.9)	8(18.6)	12(26.7)		P3 = 0.357
Blood pressure among studied cases						
Systolic blood pressure (mmHg)	Before	133.18 ± 14.22	133.95 ± 15.29	134.8 ± 15.62	F = 0.131 *p* = 0.878	P1 = 0.809 P2 = 0.610 P3 = 0.792
	After	132.13 ± 12.79	128.81 ± 13.54	132.67 ± 14.26	F = 1.04 *p* = 0.357	P1 = 0.253 P2 = 0.852 P3 = 0.185
*p*-value		0.001 *	0.001 *	0.001 *		
Diastolic blood pressure (mmHg)	Before	84.11 ± 8.21	84.37 ± 8.66	85.69 ± 9.32	F = 0.419 *p* = 0.658	P1 = 0.889 P2 = 0.394 P3 = 0.481
	After	84.29 ± 7.57	82.49 ± 7.17	84.8 ± 7.90	F = 1.13 *p* = 0.327	P1 = 0.266 P2 = 0.749 P3 = 0.154
*p*-value		0.749	0.001 *	0.07		
Anthropometric measurements of the studied groups before and after treatment						
Height (cm)	Before	168.09 ± 8.15	168 ± 8.19	165.38 ± 9.15	F = 1.46 *p* = 0.235	P1 = 0.961 P2 = 0.133 P3 = 0.151
Weight (kg)	Before	95.51 ± 15.22	91.53 ± 15.80	94.36 ± 14.08	F = 0.809 *p* = 0.447	P1 = 0.217 P2 = 0.716 P3 = 0.381
	After	95.2 ± 16.67	86.74 ± 16.08	91.87 ± 13.06	F = 3.38 *p* = 0.037 *	P1 = 0.01 * P2 = 0.304 P3 = 0.120
*p*-value		0.666	0.001 *	0.005 *		
BMI (kg/m^2^)	Before	33.78 ± 4.85	32.70 ± 6.21	34.60 ± 5.23	F = 1.34 *p* = 0.265	P1 = 0.353 P2 = 0.479 P3 = 0.105
	After	33.63 ± 5.17	30.92 ± 6.41	33.69 ± 4.89	F = 3.59 *p* = 0.03 *	P1 = 0.023 * P2 = 0.959 P3 = 0.02 *
*p*-value		0.545	0.001 *	0.007 *		
Waist circumference (WC) (cm)	Before	111.44 ± 11.61	108.91 ± 12.85	114.38 ± 10.0	F = 2.48 *p* = 0.087	P1 = 0.304 P2 = 0.230 P3 = 0.028 *
	After	111.93 ± 13.08	103.60 ± 13.95	112.98 ± 9.62	F = 7.57 *p* = 0.001 *	P1 = 0.002 * P2 = 0.689 P3 = 0.001 *
*p*-value		0.357	0.001 *	0.07		
Hip circumference (HC)(cm)	Before	119.78 ± 11.19	117.33 ± 13.33	123.24 ± 9.45	F = 3.0 *p* = 0.053	P1 = 0.315 P2 = 0.152 P3 = 0.016 *
	After	120.07 ± 12.08	114.28 ± 14.49	122.60 ± 9.52	F = 5.38 *p* = 0.006 *	P1 = 0.027 * P2 = 0.325 P3 = 0.325
*p*-value		0.569	0.001 *	0.125		
Waist–hip ratio (WHR)	Before	0.931 ± 0.05	0.928 ± 0.06	0.928 ± 0.038	F = 0.056 *p* = 0.946	P1 = 0.812 P2 = 0.749 P3 = 0.937
	After	0.930 ± 0.053	0.906 ± 0.06	0.921 ± 0.04	F = 2.66 *p* = 0.073	P1 = 0.024 * P2 = 0.41 P3 = 0.145
*p*-value		0.846	0.001 *	0.038 *		
Waist–stature ratio (WSR)	Before	0.663 ± 0.07	0.652 ± 0.09	0.696 ± 0.07	F = 4.01 *p* = 0.02 *	P1 = 0.495 P2 = 0.04 * P3 = 0.008 *
	After	0.666 ± 0.07	0.619 ± 0.098	0.685 ± 0.07	F = 7.65 *p* = 0.001 *	P1 = 0.007 * P2 = 0.277 P3 = 0.001 *
*p*-value		0.211	0.001 *	0.019 *		
Midarm circumference (MUAC)	Before	36.41 ± 3.74	35.53 ± 4.06	36.54 ± 3.84	F = 0.874 *p* = 0.420	P1 = 0.291 P2 = 0.871 P3 = 0.224
	After	36.42 ± 4.41	34.03 ± 4.02	35.80 ± 3.62	F = 4.13 *p* = 0.018 *	P1 = 0.006 * P2 = 0.465 P3 = 0.042 *
*p*-value		0.788	0.001 *	0.002 *		

F: One Way ANOVA test, P1: difference between control group 1 vitamin E (VE), and N-acetyl cysteine (NAC) group2, P2: difference between control group 1 and Rosuvastatin (RSV) group 3, P3: difference between NAC group2 and RSV group 3, MC: Monte Carlo correction for Chi-Square test, * statistically significant, For comparison of before and after treatment (Paired *t* test). Data expressed as mean ± SD.

**Table 2:** Steatosis and fibrosis degree and studied markers among the studied groups before and after treatment.

		Control Group (VE) *n* = 45	NAC Group *n* = 43	RSV Group *n* = 45	Test of Significance	Intergroup Significance
Steatosis	Before	302.96 ± 44.82	309.23 ± 48.96	315.31 ± 44.57	F = 0.807 *p* = 0.448	P1 = 0.525 P2 = 0.206 P3 = 0.538
	S1 S2 S3	8(17.8) 12(26.7) 25(55.6)	8(18.6) 11(25.6) 24(55.8)	6(13.3) 9(20.0) 30(66.7)		
	After	284.62 ± 52.84	265.33 ± 65.16	291.60 ± 47.99	F = 2.62 *p* = 0.08	P1 = 0.106 P2 = 0.553 P3 = 0.029 *
	S0 S1 S2 S3	4(8.9) 5(11.1) 12(26.7) 24(53.3)	12(27.9) 6(14) 7(16.3) 18(41.9)	5(11.1) 1(2.2) 13(28.9) 26(57.8)		
*p*-value		0.017 *	0.001 *	0.004 *		
Fibrosis	Before	5.9(4.4–8.85)	5.6(4.9–6.4)	5.5(4.05–7.35)	KW = 0.571 *p* = 0.752	P1 = 0.679 P2 = 0.413 P3 = 0.881
	F0 F1 F2 F3 F4	19(42.2) 8(17.8) 11(24.4) 2(4.4) 5(11.1)	20(46.5) 16(37.2) 3(7) 3(7) 1(2.3)	21(46.7) 12(26.7) 6(13.3) 1(2.2) 5(11.1)		
	After	5.6(4.35–8.20)	4.9(4.4–5.9)	6(4.55–7.6)	KW = 5.81 *p* = 0.06	P1 = 0.051 P2 = 0.987 P3 = 0.028 *
	F0 F1 F2 F3 F4	22(48.9) 9(20.0) 7(15.6) 2(4.4) 5(11.1)	31(72.1) 7(16.3) 4(9.3) 0 1(2.3)	21(46.7) 12(26.7) 5(11.1) 2(4.4) 5(11.1)		
*p*-value		0.977	0.001 *	0.218		
FAST score	Before	0.370(0.325–0.54)	0.38(0.3–0.5)	0.37(0.265–0.495)	KW = 1.30 *p* = 0.522	P1 = 0.520 P2 = 0.276 P3 = 0.562
	After	0.26(0.18–0.425)	0.14(0.09–0.22)	0.26(0.12–0.36)	KW = 17.56 *p* = 0.001 *	P1 = 0.001 * P2 = 0.188 P3 = 0.007 *
*p*-value		0.001 *	0.001 *	0.001 *		
FNI	Before	0.510(0.315–0.605)	0.520(0.36–0.59)	0.43(0.315–0.56)	KW = 2.43 *p* = 0.297	P1 = 0.884 P2 = 0.196 P3 = 0.164
	After	0.25(0.18–0.425)	0.17(0.12–0.29)	0.24(0.09–0.34)	KW = 7.90 *p* = 0.019 *	P1 = 0.007 * P2 = 0.039 * P3 = 0.622
*p*-value		0.001 *	0.001 *	0.001 *		
FIB-4	Before	1.4(1.06–2.26)	1.11(0.73–1.37)	1.37(1.09–1.92)	KW = 10.79 *p* = 0.005 *	P1 = 0.004 * P2 = 0.693 P3 = 0.001 *
	After	1.41(0.875–1.85)	0.81(0.60–0.98)	1.26(0.975–1.58)	KW = 23.50 *p* = 0.001 *	P1 = 0.001 * P2 = 0.196 P3 = 0.164
*p*-value		0.413	0.001 *	0.04 *		
MACK-3	Before	0.358(0.273–0.421)	0.332(0.267–0.445)	0.263(0.182–0.364)	KW = 8.71 *p* = 0.013 *	P1 = 0.780 P2 = 0.007 * P3 = 0.018 *
	After	0.329(0.265–0.433)	0.253(0.222–0.380)	0.280(0.197–0.377)	KW = 4.38 *p* = 0.112	P1 = 0.043 * P2 = 0.117 P3 = 0.838
*p*-value		0.059	0.001 *	0.046 *		

F: One Way ANOVA test; KW: Kruskal–Wallis test; P1: difference between control and NAC groups; P2: difference between control and RSV groups; P3: difference between NAC group 2 and RSV group 3; * statistically significant for comparison of before and after treatment (Paired *t* test, Wilcoxon signed rank test); data expressed as mean ± SD or median (interquartile range).

## Data Availability

Data will be provided upon reasonable request to the corresponding author.

## Supplementary Materials

The following supporting information can be downloaded at https://www.mdpi.com/article/10.3390/ph18050650/s1; Table S1: Laboratory values of the studied groups before and after treatment; Table S2: Common Terminology Criteria for Adverse Events, Version 5.00 (CTCAE).

## Abbreviations

AASLD; The American Association for the Study of Liver Diseases, ALP; Alkaline phosphatase, ALT; alanine aminotransferase, AST; aspartate aminotransferase, BMI; body mass index, CAP; controlled attenuation parameter, CBC; complete blood count, CK18; cytokeratin-18, CONSORT; Consolidated Standards of Reporting Trials, CPK; creatine phosphokinase, CTCAE; Common Terminology Criteria for Adverse Events, Version 5.00, CYP; Cytochromes P, ECM; extra-cellular matrix, eNOS; endothelial nitric oxide synthase, ER; endoplasmic reticulum, ERK; extracellular signal-regulated kinase, FAST; fibroscan-aspartate aminotransferase score, FBG; fasting blood glucose, FGF21; fibroblast growth factor-21, FIB-4; the Fibrosis-4 Index, FNI; fibrotic NASH index, GDF15; growth differentiation factor 15, GGT; Gam-ma-glutamyl Transferase, GPx; glutathione peroxidase, GSH; glutathione, GSS; glutathione synthase, GR; glutathione reductase, GST; glutathione S-transferase; Grps; groups, HC; Hip circumference, HDL; high-density lipoprotein, HbA1c; hemoglobin A1c, HFD; high-fat diet, HSC; hepatic stellate cells, HMG-CoA; 3-Hydroxy-3-Methylglutaryl-Coenzyme, HO-1; heme oxygenase 1, HOMA-IR; homeostatic model assessment for insulin resistance, HRQoL; health-related quality of life, HSC; hepatic stellate cells, IFN-γ; interferon gamma, IL-6; interleukin 6, IR; insulin resistance, LDL; low-density lipoprotein, LSM; liver stiffness measurement, MACK-3; combination of aspartate aminotransferase, homeostasis model assessment, and cytokeratin 18, MDA; malondialdehyde, MMPs; matrix metalloproteinases, MUAC; mid-arm circumference, NAC; N-acetyl cysteine, NAFLD; nonalcoholic fatty liver disease, NASH; non-alcoholic steatohepatitis, NF-kB; nuclear factor-kB, NLRP3; NOD-like receptor-associated protein 3, NOX1; NADPH oxidase enzyme1, Nrf2; Nuclear factor erythroid 2–related factor 2, OS; oxidative stress, PI3K/Akt; the phosphatidylinositol 3-kinase (PI3K)/protein kinase B (AKT)/mammalian target of rapamycin (mTOR), PIIINP; N-terminal propeptide of procollagen type III, PIVENS Trial; the pioglitazone versus vitamin E versus placebo for the treatment of non-diabetic patients with NASH, PPARα; peroxisome proliferator-activated receptor-α, PPARs; Peroxisome proliferator-activated receptor, ROS; Reactive Oxygen Species, RSV; Rosuvastatin, SF-36; Short-Form 36, SOD; superoxide dismutase, TβRIII; type III transforming growth factor β (TGF-β) receptor, TBIL; total bilirubin, TGF-β; transforming growth factor beta, TIMP-1; tissue inhibitor of metalloproteinases 1, TNF-α; tumor necrosis factor-alpha, TRX; thioredoxin, TXNIP; thioredoxin-interacting protein, VE; vitamin E, VLDL; very low-density lipoprotein, WC; waist circumference, WHR; waist–hip ratio, WSR; waist–stature ratio, α-SMA; α-smooth muscle actin, *α*-TOH; alpha-tocopherol.
