# Supplementary material for: A Comparative Study of N-Acetyl Cysteine, Rosuvastatin, and Vitamin E in the Management of Patients with Non-Alcoholic Steatohepatitis: A Randomized Controlled Trial
Source: Pharmaceuticals (Basel). 2025 Apr 29;18(5):650. doi: 10.3390/ph18050650 (PMC12114936; doi:10.3390/ph18050650)
Supplement: Supplementary file 1 [file pharmaceuticals-18-00650-s001.zip › pharmaceuticals-3479004-supplementary.pdf]

Supplemental Digital Content 1. Laboratory values of the studied groups before and after treatment

		Control group (VE) N=45	NAC group N=43	RSV group N=45	Test of significance	Intergroup significance
**Platelet**	Before	213.78±55.44	239.95±64.78	219.27±56.58	F=2.39<br>P=0.095	P1=0.039*<br>P2=0.660<br>P3=0.103
	After	208.11±52.59	259.05±47.32	217.98±55.66	F=11.77<br>P=0.001*	P1=0.001*<br>P2=0.370<br>P3=0.001*
**P-value**		0.413	0.001*	0.04*		
**ALT**	Before	48(38.75-75)	54(42-76)	45(39-58)	KW=4.98<br>P=0.083	P1=0.559<br>P2=0.131<br>P3=0.028*
	After	45(37-60)	45(36-52)	42(32-51)	KW=2.97<br>P=0.227	P1=0.582<br>P2=0.099<br>P3=0.241
**P-value**		0.01*	0.002*	0.02*		
**AST**	Before	38(24.3-55.5)	31(22-43)	28(22-43)	KW=7.0<br>P=0.03*	P1=0.104<br>P2=0.009*<br>P3=0.358
	After	33.7(27.8-39.7)	26(20-34)	28(22-37)	KW=9.0<br>P=0.01*	P1=0.005*<br>P2=0.03*<br>P3=0.354
**P-value**		0.076	0.005*	0.916		
**AST/ALT**	Before	0.88(0.695-1.05)	0.84(0.61-1.1)	0.86(0.74-1.15)	KW=1.63<br>P=0.443	P1=0.388<br>P2=0.695<br>P3=0.213
	After	0.94(0.74-1.13)	0.85(0.7-1.05)	1.06(0.835-1.24)	KW=6.48<br>P=0.039*	P1=0.291<br>P2=0.151<br>P3=0.01*
**P-value**		0.434	0.109	0.047*		
**Alkaline phosphatase (ALP)**	Before	110.87±34.49	106.16±31.05	125.04±18.21	F=5.18<br>P=0.007*	P1=0.444<br>P2=0.02*<br>P3=0.003*
	After	107.82±33.53	105.18±28.95	106.99±16.38	F=0.108<br>P=0.898	P1=0.650<br>P2=0.885<br>P3=0.756
**P-value**		<0.001*	<0.001*	<0.001*		
**Total bilirubin (TBIL)**	Before	0.8(0.58-1.06)	0.77(0.60-0.96)	0.73(0.6-0.95)	Kw=0.418<br>P=0.811	P1=0.664<br>P2=0.526<br>P3=0.861
	After	0.71(0.595-1)	0.61(0.42-0.81)	0.70(0.555-0.935)	Kw=8.48<br>P=0.014*	P1=0.005*<br>P2=0.298<br>P3=0.054
**P-value**		0.012*	<0.001*	<0.001*		
**Gamma-glutamyl transferase (GGT)**	Before	21.5(15.3-28.25)	25(21-29)	26.8(21-33)	Kw=8.28<br>P=0.016*	P1=0.048*<br>P2=0.006*<br>P3=0.343
	After	23.61(17.5-30.35)	20(17-25)	25(19.39-31)	Kw=8.33<br>P=0.016*	P1=0.053<br>P2=0.480

						P3=0.004*
**P-value**		<0.001*	<0.001*	<0.001*		
**Blood urea**	Before	32.54±8.58	28.92±8.0	31.11±9.38	F=1.94<br>P=0.148	P1=0.053<br>P2=0.435<br>P3=0.239
	After	31.09±7.92	25.14±7.95	32.60±9.44	F=9.47<br>P=0.001*	P1=0.001*<br>P2=0.40<br>P3=0.001*
**P-value**		<0.001*	0.002*	<0.001*		
**Serum creatinine**	Before	0.89(0.725-1.1)	0.81(0.7-0.98)	0.89(0.74-0.985)	Kw=2.98<br>P=0.225	P1=0.081<br>P2=0.513<br>P3=0.312
	After	0.6(0.72-0.925)	0.63(0.5-0.83)	0.8(0.675-0.925)	Kw=6.14<br>P=0.046*	P1=0.061<br>P2=0.390<br>P3=0.025*
**P-value**		<0.001*	<0.001*	<0.001*		
**Uric acid**	Before	6.2(4.95-7.0)	5.3(4.1-6)	5.4(4.45-6.25)	Kw=10.40<br>P=0.006*	P1=0.003*<br>P2=0.01*<br>P3=0.628
	After	5(4.15-5.85)	3.9(3.2-4.7)	5(4.24-5.86)	Kw=19.43<br>P=0.001*	P1=0.001*<br>P2=0.750<br>P3=0.001*
**P-value**		<0.001*	<0.001*	<0.001*		
**Creatine phosphokinase (CPK)**	Before	100.6(73.5-131.25)	70.5(55-136.7)	110.3(100.9-145.35)	KW=15.94<br>P=0.001*	P1=0.251<br>P2=0.002*<br>P3=0.001*
	After	92(62.5-133.3)	57.8(43-115)	115(102.4-148.5)	KW=33.38<br>P=0.001*	P1=0.001*<br>P2=0.005*<br>P3=0.001*
**P-value**		0.001*	0.001*	0.001*		
**HBA1C**	Before	5.73±0.44	5.80±0.49	5.65±0.58	F=0.985<br>P=0.376	P1=0.50<br>P2=0.464<br>P3=0.163
	After	5.69±0.46	5.69±0.60	5.65±0.61	F=0.09<br>P=0.632	P1=0.998<br>P2=0.713<br>P3=0.718
**P-value**		0.295	0.004*	0.770		
**Fasting blood glucose (FBG)**	Before	99.63±12.24	101.74±11.06	101.69±12.20	F=0.460<br>P=0.632	P1=0.405<br>P2=0.412<br>P3=0.983
	After	100.78±12.72	98.08±13.26	107.94±15.04	F=6.10<br>P=0.003*	P1=0.357<br>P2=0.015*<br>P3=0.001*
**P-value**		0.935	0.009*	0.003*		
**Insulin**	Before	13.21±1.63	13.61±1.54	11.39±2.13	F=19.38<br>P=0.001*	P1=0.299<br>P2=0.001*<br>P3=0.001*
	After	12.96±1.91	12.44±2.13	12.20±2.75	F=1.29<br>P=0.278	P1=0.287<br>P2=0.118<br>P3=0.628
**P-value**		0.266	0.001*	0.004*		
**HOMA-IR**	Before	3.26±0.65	3.42±0.58	2.88±0.73	F=7.58<br>P=0.001*	P1=0.267<br>P2=0.008*

						P3=0.001*
	After	3.23±0.65	3.04±0.795	3.17±0.72	F=0.773<br>P=0.464	P1=0.229<br>P2=0.728<br>P3=0.369
**P-value**		0.407	0.001*	0.008*		
**Serum cholesterol**	Before	195.47±32.61	193.88±49.51	214.26±39.79	F=3.39<br>P=0.037*	P1=0.857<br>P2=0.032*<br>P3=0.02*
	After	194.05±34.36	183.40±49.54	171.25±30.28	F=3.89<br>P=0.023*	P1=0.20<br>P2=0.006*<br>P3=0.144
**P-value**		0.484	0.001*	0.001*		
**Triglycerides (TGs)**	Before	132(106.2-156.4)	150(108-202)	153(123.5-198)	Kw=5.36<br>P=0.069	P1=0.082<br>P2=0.025*<br>P3=0.987
	After	126(95.85-150.5)	136(97-174)	116(93.5-149.65)	Kw=3.52<br>P=0.171	P1=0.274<br>P2=0.448<br>P3=0.06
**P-value**		0.843	0.001*	0.001*		
**LDL**	Before	119.33±26.92	120.57±38.24	127.80±41.68	F=0.719<br>P=0.489	P1=0.872<br>P2=0.268<br>P3=0.349
	After	115.89±24.79	112.74±40.46	96.73±34.72	F=4.13<br>P=0.018*	P1=0.663<br>P2=0.008*<br>P3=0.028*
**P-value**		0.001*	0.001*	0.001*		
**VLDL**	Before	29(21.5-38)	34(26-43)	42(37-47.7)	Kw=27.98<br>P=0.001*	P1=0.133<br>P2=0.001*<br>P3=0.001*
	After	29(18.5-38.5)	27(20.8-35)	31(27.4-36.9)	Kw=5.48<br>P=0.07	P1=0.828<br>P2=0.106<br>P3=0.018*
**P-value**		0.258	0.001*	0.001*		
**HDL**	Before	43.16±10.12	40.77±10.56	40.32±8.47	F=1.09<br>P=0.336	P1=0.253<br>P2=0.169<br>P3=0.829
	After	44.88±10.18	43.99±11.18	46.35±10.32	F=0.562<br>P=0.571	P1=0.694<br>P2=0.510<br>P3=0.297
**P-value**		0.001*	0.001*	0.001*		
**Cholesterol/HDL**	Before	4.78±1.41	4.99±1.61	5.48±1.37	F=2.69<br>P=0.07	P1=0.491<br>P2=0.025*<br>P3=0.123
	After	4.55±1.32	4.37±1.45	3.82±0.88	F=4.18<br>P=0.01*	P1=0.507<br>P2=0.006*<br>P3=0.04*
**P-value**		0.001*	0.001*	0.001*		
**LDL/HDL**	Before	2.77(2.06-3.44)	3.21(2.1-4.33)	3.03(2.48-4.13)	KW=1.43<br>P=0.489	P1=0.442<br>P2=0.237<br>P3=0.710

	After	2.68(2.03-3.13)	2.71(1.64-3.86)	1.97(1.58-2.56)	KW=10.06<br>P=0.007*	P1=0.993<br>P2=0.001*<br>P3=0.037*
**P-value**		0.001*	0.001*	0.001*		

*F: One Way ANOVA test, KW: Kruskal Wallis test P1: difference between control and NAC groups, P2: difference between control and RSV groups, P3: difference between difference between NAC and RSV groups*statistically significant For comparison of before and after treatment (Paired t test , Wilcoxon signed rank test), Data expressed as mean±SD or median (interquartile range)*

Supplemental Digital Content 2. Common Terminology Criteria for Adverse Events, Version 5.00 (CTCAE)

**Adverse Events**	**Grade**	**Control group 1 (VE) N=45**	
		N	%
**Blurred vision**	I	2	4.4
**Diarrhea**	I<br>II	8<br>1	17.8%<br>2.2%
**Dizziness**	I	5	11.1%
**Headache**	I	8	17.8%
**Nausea**	I	9	20.0%
**Stomach pain**	I	6	13.3%
**Fatigue**	I<br>II	4<br>1	8.9%<br>2.2%
		**NAC group 2 N=43**	
		N	%
**Allergic reaction (Grade I)**	I	2	4.7%
**Pruritis**	I	3	7.0%
**Flushing**	I	3	7.0%
**Adult respiratory distress syndrome**	I	2	4.7%
**Hypotension**	I	3	7.0%
**Dyspepsia**	I<br>II	5<br>2	11.6%<br>4.7%
**Stomach pain**	I	4	9.3%
**Nausea**	I<br>II	6<br>2	14.0%<br>4.7%
**Vomiting**	I	4	9.3%
**Fatigue**	I	2	4.7%

		RSV group 3<br>N=45	
		N	%
**Myalgia**	I	10	22.2%
**Hyperglycemia**			
	I	2	4.4%
	II	2	4.4%
**Constipation**	I	4	8.9%
**Nausea**			
	I	4	8.9%
	II	1	2.2%
**T. bilirubin**	I	1	2.2%
**Dizziness**	I	2	4.4%
**Headache**	I	5	11.1%
**Arthralgia**	I	3	6.7%
